# Effects of calcium channel blockers in patients with heart failure with preserved ejection fraction: A protocol for systematic review and meta-analysis

**DOI:** 10.1371/journal.pone.0307258

**Published:** 2024-08-19

**Authors:** Hidekatsu Fukuta, Toshihiko Goto, Takeshi Kamiya

**Affiliations:** 1 Core Laboratory, Nagoya City University Graduate School of Medical Sciences, Nagoya, Japan; 2 Department of Cardiology, Nagoya City University Graduate School of Medical Sciences, Nagoya, Japan; 3 Department of Medical Innovation, Nagoya City University Graduate School of Medical Sciences, Nagoya, Japan; Kurume University School of Medicine, JAPAN

## Abstract

**Background:**

Nearly half of patients with heart failure (HF) have preserved ejection fraction (EF) and the mortality and morbidity of patients with HF with preserved EF (HFpEF) are high. Patients with HFpEF are often elderly and their primary chronic symptom is severe exercise intolerance. Due to the frequent coexistence of hypertension in HFpEF patients, the use of anti-hypertensive medications is common in their treatment. While many cohort studies and several randomized controlled trials (RCTs) have examined the effectiveness of various anti-hypertensive drugs such as beta-blockers and renin-angiotensin system inhibitors in HFpEF, the role of calcium channel blockers (CCBs) remains uncertain. Despite several RCTs and cohort studies exploring the effects of CCBs on prognosis and exercise capacity in HFpEF patients, the findings have been inconsistent, likely due to limited statistical power and/or variations in study design. Therefore, our aim is to conduct a systematic review and meta-analysis of studies on the effects of CCBs in these patients.

**Methods:**

This meta-analysis will include RCTs and cohort studies on the effect of CCBs in HFpEF patients. Information of studies will be collected from PubMed, Web of Science, and Scopus. The primary outcome of interest will be prognosis. The secondary outcome of interest will be exercise capacity.

**Discussion:**

Synthesizing our meta-analytical results with expert consensus could contribute to the formulation of updated clinical guidelines. Our systematic review and meta-analysis will provide directions for future research on the use of CCBs in HFpEF patients.

**Systematic review registration:**

INPLASY202430097.

## Introduction

Nearly half of patients with heart failure (HF) in the community have preserved ejection fraction (EF) and the mortality and morbidity of patients with HF with preserved EF (HFpEF) are high [1–4]. Patients with HFpEF are often elderly and their primary chronic symptom is severe exercise intolerance that results in a reduced quality of life [5, 6].

Sodium-glucose cotransporter 2 inhibitors are currently recommended as a class I indication for HFpEF patients [7]. Based on large, high-quality randomized controlled trials (RCTs), angiotensin receptor neprilysin inhibitors and mineralocorticoid receptor antagonists are the consensus treatment for HFpEF patients [8, 9]. Non-dihydropyridine calcium channel blockers (CCBs) are often considered first-line agents for heart rate control in HFpEF patients with atrial fibrillation [10]. Due to the frequent coexistence of hypertension in HFpEF patients, the use of anti-hypertensive medications is common in their treatment [11]. While many cohort studies and several RCTs have examined the effectiveness of various anti-hypertensive drugs such as renin-angiotensin system inhibitors (angiotensin converting enzyme inhibitors and angiotensin receptor blockers) and beta-blockers in HFpEF patients [12–17], the role of CCBs remains uncertain. Despite several RCTs and cohort studies exploring the effects of CCBs on prognosis and exercise capacity in HFpEF, the findings have been inconsistent, likely due to limited statistical power and/or variations in study design [18–24]. Specifically, significant improvement in exercise capacity with CCB treatment was reported in one RCT [19], but in another RCT, such improvement was not observed [18]. An association between CCB use and better survival was observed in one cohort study [21], but in other cohort studies, such an association was not observed [20, 22]. These discrepancies highlight gaps such as small sample sizes, differing patient populations, and variations in CCB subclasses used. Thus, there is a critical need to comprehensively synthesize existing evidence. Therefore, our aim is to conduct a systematic review and meta-analysis of studies on the effects of CCBs in these patients.

## Methods

This study has been registered on the International Platform of Registered Systematic Review and Meta-analysis Protocols with registration number of INPLASY202430097 (DOI: 10.37766/inplasy2024.3.0097). This protocol for meta-analysis will be performed according to the Preferred Reporting Items for Systematic Review and Meta-analysis Protocols (PRISMA-P) statement [25].

### Search strategy

The electronic databases for the literature search will include PubMed, Web of Science, and Scopus. For search of the eligible studies, the following keywords and Medical Subject Heading will be used:

#1 “heart failure with preserved ejection fraction” OR “heart failure with normal ejection fraction” OR “diastolic heart failure”

#2 “calcium channel blockers” OR “calcium channel antagonists”

#3 “prognosis” OR “death” OR “mortality” OR “hospitalization” OR “outcomes”

#4 “exercise capacity” OR “functional capacity” OR “exercise intolerance” OR “oxygen consumption” OR “oxygen uptake” OR “walk distance” OR “walk test” OR “quality of life”

#5 #1 AND #2 AND #3 (primary outcome)

#6 #1 AND #2 AND #4 (secondary and other outcomes)

**Fig 1:**
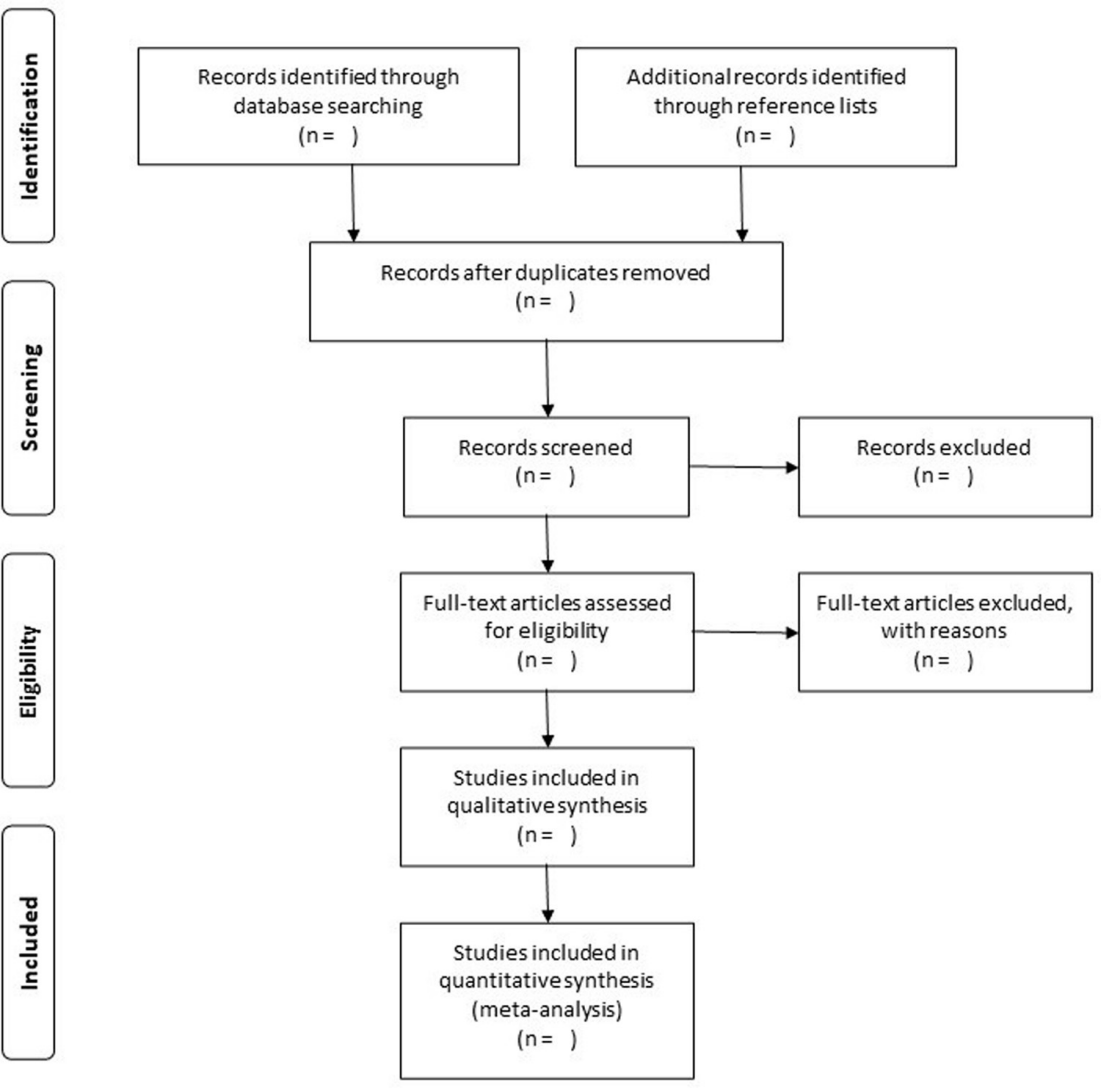
PRISMA flow diagram.

Literature search will also be conducted by manual screening of reference lists of relevant reviews and retrieved articles. Two researchers (HF and TK) will independently perform the literature search and will extract study-level data from included studies. Disagreements will be resolved by consensus. The literature search will be repeated before completing the data extraction, and potential new studies published during the work process will be added to the result. Study selection will be conducted in a PRISMA-compliant flow chart (Fig 1). Only articles published in the English language will be included. The literature search will be started in Jun 2024 and the data extraction will be completed by September 2024.

### Study design

RCTs and prospective and retrospective cohort studies will be included. Case–control studies will be excluded.

### Selection criteria

Inclusion criteria for this meta-analysis will be: (1) include HFpEF patients treated with CCBs; (2) compare between CCBs and controls; and (3) assess prognosis, exercise capacity, and/or quality of life.

### Outcomes

The primary outcome of interest will be prognosis, including death from cardiovascular causes, hospitalization for HF, and all-cause death. The secondary outcome of interest will be exercise capacity, as assessed by peak oxygen uptake [26], 6-minute walk distance [27], and exercise time [28]. Other outcome of interest will be health-related quality of life, as assessed by the scores of the Minnesota Living With Heart Failure Questionnaire [29] and the Kansas City Cardiomyopathy Questionnaire [30] and the Congestive Heart Failure score [31].

### Data extraction

Two reviewers (HF and TK) will independently extract relevant data from retrieved studies, including author, study design, study time, country, number of participants, baseline characteristics (age, sex, comorbidities), clinical outcomes (prognosis, exercise capacity, health related-quality of life), and information on the methodological quality (selection of cohorts, assessment of outcome, etc). Disagreements will be resolved by consensus. We will contact the corresponding author of eligible studies when insufficient information is available to perform our meta-analysis.

### Quality assessment

The Cochrane Risk of Bias tool will be used to assess quality of RCTs included in the study [32]. The quality of cohort studies will be evaluated by the Newcastle-Ottawa Scale tool (http://www.ohri.ca/programs/clinical_epidemiology/oxford.asp). The quality of evidence for the outcomes will be evaluated by use of the Grading of Recommendations Assessment, Development and Evaluation (GRADE) system [33]. The quality of evidence will be evaluated across the domains of risk of bias, consistency, directness, precision, and publication bias.

### Statistical analysis

For mortality and morbidity, hazard ratios will be pooled. For continuous outcomes, the effect size for the intervention will be calculated by the difference between the means of the intervention and control groups at the end of the intervention. If the outcome is measured on the same scale, the weighted mean difference and 95% confidence interval (CI) will be calculated. Otherwise, the standardized mean difference and 95% CI will be calculated. For each outcome, heterogeneity will be assessed using the Cochran’s Q and *I*^2^ statistic; for the Cochran’s Q and *I*^2^ statistic, a p value of <0.1 and *I*^2^>50%, will be considered significant, respectively. When there is significant heterogeneity, the data will be pooled using a random-effects model, otherwise a fixed-effects model will be used. When there are more than 10 studies included, publication bias will be assessed graphically using a funnel plot and mathematically using Egger test. For these analyses, Comprehensive Meta Analysis Software version 2 (Biostat, Englewood, NJ, USA) and STATA 16 software (Stata Corp LP, TX, USA) will be used.

### Sensitivity analysis

Subgroup analysis stratified by CCB subclass (dihydropyridine or non-dihydropyridine CCBs), study design (RCT or cohort study), and baseline clinical characteristics (atrial fibrillation and coronary artery disease) will be performed. Meta-regression will be used to determine whether the effect of CCBs will be confounded by baseline clinical characteristics such as age, sex, New York Heart Association (NYHA) functional class, atrial fibrillation, and coronary artery disease. Based on an earlier report, when the number of included studies reporting the primary outcome in the present meta-analysis is less than 5, sensitivity analysis will not be performed [34].

### Ethical issues

This meta-analysis is a literature study. Ethical approval is not required because this meta-analysis will not involve any subject directly.

## Discussion

The use of antihypertensive medications is common among HFpEF patients, owing to the frequent coexistence of hypertension in this population [11]. Numerous cohort studies and several RCTs have investigated the effects of various antihypertensive agents, such as renin-angiotensin system inhibitors (angiotensin converting enzyme inhibitors and angiotensin receptor blockers) and beta-blockers in HFpEF patients [12–17]. Specifically, in our prior meta-analysis, encompassing 12 cohort studies (involving 26,557 patients) and 3 RCTs (enrolling 8001 patients), we observed a potential mortality benefit associated with RAS inhibitors in HFpEF in cohort studies, while such benefit was not observed in RCTs [13]. Another meta-analysis of 7 RCTs, involving 569 patients, did not demonstrate an improvement in exercise capacity, as assessed by the 6-minute walk distance, with RAS inhibitor treatment in HFpEF [14]. Similarly, our meta-analysis involving 11 observational cohort studies (enrolling 27,590 patients) and 3 RCTs (involving 1046 patients) suggested a potential mortality benefit of beta-blockers in HFpEF in cohort studies but not in RCTs [16]. Another meta-analysis of 5 RCTs, including 538 patients, did not indicate a clear beneficial effect of beta-blockers on HF severity, as assessed by NYHA functional class, exercise capacity, and plasma brain natriuretic peptide levels in HFpEF patients [17]. However, the role of CCBs in HFpEF patients remains uncertain.

Only one meta-analysis has been conducted on the effects of CCBs in HFpEF [35]. The meta-analysis examined 2 RCTs and 3 cohort studies including 11,208 patients and reported the potential benefit of CCBs for all-cause mortality and hospitalization. However, we consider this meta-analysis limited for several reasons. First, the analysis failed to stratify between dihydropyridine and non-dihydropyridine CCBs, despite their distinct pharmacological profiles; dihydropyridine CCBs tend to be more potent vasodilators than non-dihydropyridine CCBs, whereas the latter have more marked negative inotropic effects [36]. Given these differences, separate analyses for each class of drugs are warranted. Additionally, the meta-analysis did not perform a separate analysis for RCTs and cohort studies. Second, based on our preliminary search, it overlooked multiple RCTs and cohort studies pertinent to the topic [18–21]. Additionally, a significant observational analysis of pooled data from large HFpEF trials, including the I-Preserve, TOPCAT, PARAGON-HF, and DELIVER trials, has been published since the completion of the meta-analysis [24] and it is inconsistent with the meta-analysis [35]. Furthermore, the meta-analysis examined the effect of CCBs only on prognosis but not on exercise capacity.

To the best of our knowledge, our meta-analysis represents the first attempt to examine the effects of CCBs on both prognosis and exercise capacity, separately considering CCB subclass and study design in HFpEF patients. Recent studies have underscored the heterogeneity in biological phenotypes within HFpEF, which traditional inclusion criteria based on left ventricular EF, B-type natriuretic peptide levels, and HF history may not fully capture [37]. This diversity poses a challenge in translating meta-analytical findings into clinical practice effectively. However, we believe that we can propose several strategies for utilizing the findings from our meta-analysis in clinical settings. First, given the recognized heterogeneity, we will conduct subgroup analyses based on emerging biological phenotypes identified in recent literature. This approach aims to identify specific HFpEF subgroups that may benefit most from CCBs, thereby providing clinicians with tailored treatment insights. Second, synthesizing our meta-analytical results with expert consensus could contribute to the formulation of updated clinical guidelines. These guidelines would aim to provide evidence-based recommendations on the use of CCBs in HFpEF patients, considering the detailed patient profiles identified through our meta-analysis. Finally, our meta-analysis will provide directions for future research on the use of CCBs in HFpEF patients.

## Abbreviations

CCBs: calcium channel blockers
EF: ejection fraction
GRADE: Grading of Recommendations Assessment, Development and Evaluation
HF: heart failure
HFpEF: heart failure with preserved ejection fraction
PRISMA-P: Preferred Reporting Items for Systematic Review and Meta-analysis Protocols
RCTs: randomized controlled trials
